# Endocrine–metabolic regulation during the transition period in dairy cows: mechanisms, biomarkers, and emerging diagnostics for subclinical ketosis

**DOI:** 10.3389/fendo.2026.1799702

**Published:** 2026-04-17

**Authors:** Mohan Mondal, Tonsing Kham Suan, Pratap Laxman Gore, Lokesh Khandelwal, Roohani Sharma, Muthu Karunakaran, Hassan Shawky Mohamed Youssef, Suman Chakraborty, Adamou Akourki

**Affiliations:** 1 Animal Physiology Laboratory, ICAR- National Dairy Research Institute (ERS), Kalyani, West Bengal, India; 2 Animal Reproduction & Gynaecology Laboratory, ICAR- National Dairy Research Institute Eastern Regional Station (ERS), Kalyani, West Bengal, India; 3 Animal Reproduction Research Institute, Agriculture Research Centre (ARC), Giza, Egypt; 4 Department of Mechanical Engineering, Indian Institute of Technology Kharagpur, Kharagpur, India; 5 De´ partement des Sciences et Techniques d’Elevage, Faculte´ d’Agronomie et des Sciences de l’Environnement, Universite´ Dan Dicko Dankoulodo de Maradi, Maradi, Niger

**Keywords:** diagnostic biomarkers, endocrine regulation, hepatic metabolism, subclinical ketosis, transition dairy cows, β-hydroxybutyrate

## Abstract

The transition period from late gestation to early lactation represents a phase of marked endocrine and metabolic adjustment in dairy cows, driven by rapidly increasing energy demands associated with the onset of milk production. Alterations in circulating insulin, growth hormone, insulin-like growth factor-1, and cortisol promote nutrient partitioning toward lactation, enhance adipose tissue lipolysis, and increase the release of non-esterified fatty acids (NEFAs) into the bloodstream. When hepatic oxidative capacity and triglyceride export mechanisms are exceeded, excess NEFA uptake favors ketogenesis and accumulation of ketone bodies, particularly β-hydroxybutyrate, resulting in subclinical ketosis (SCK). Although SCK lacks overt clinical signs, it is highly prevalent during early lactation and is associated with impaired immune function, increased susceptibility to postpartum disorders, reduced milk yield, and compromised reproductive performance. This review summarizes current knowledge on the endocrine regulation of lipid mobilization, hepatic metabolic flexibility, and immunometabolic adaptations that contribute to the development of SCK in transition dairy cows. Diagnostic approaches are discussed with emphasis on established biomarkers such as NEFA and β-hydroxybutyrate, milk-based infrared spectroscopy, and emerging biosensor-based technologies, highlighting their advantages, limitations, and applicability for herd-level monitoring. The review also integrates evidence on the economic consequences of SCK and recent advances in precision nutrition, molecular profiling, and machine-learning-based prediction tools that may support earlier detection and targeted management. This synthesis is intended to provide a consolidated overview of endocrine–metabolic mechanisms and diagnostic strategies relevant to subclinical ketosis in dairy cattle during the transition period.

## Graphical Abstract

**Figure:**
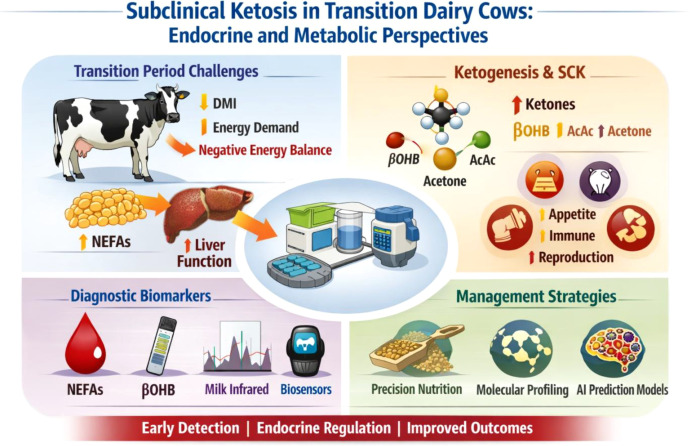
Integrated endocrine–hepatic–immune interactions underlying subclinical ketosis (SCK) in transition dairy cows. Schematic representation of the coordinated endocrine alterations, hepatic metabolic responses, and immune dysfunction contributing to subclinical ketosis. Reduced insulin, elevated growth hormone (GH), and increased cortisol enhance adipose lipolysis and elevate circulating non-esterified fatty acids (NEFAs). Excess NEFA influx exceeds hepatic oxidative capacity, resulting in increased ketogenesis (β-hydroxybutyrate, acetoacetate, acetone), triglyceride accumulation, and impaired gluconeogenesis. Accumulation of ketone bodies and free fatty acids promotes oxidative stress and leukocyte dysfunction, predisposing cows to inflammatory diseases such as metritis and mastitis. Arrows illustrate the bidirectional feedback among endocrine signaling, hepatic metabolic inflexibility, and immune impairment that collectively drive the development of SCK.

## 1 Introduction

**Figure 1:**
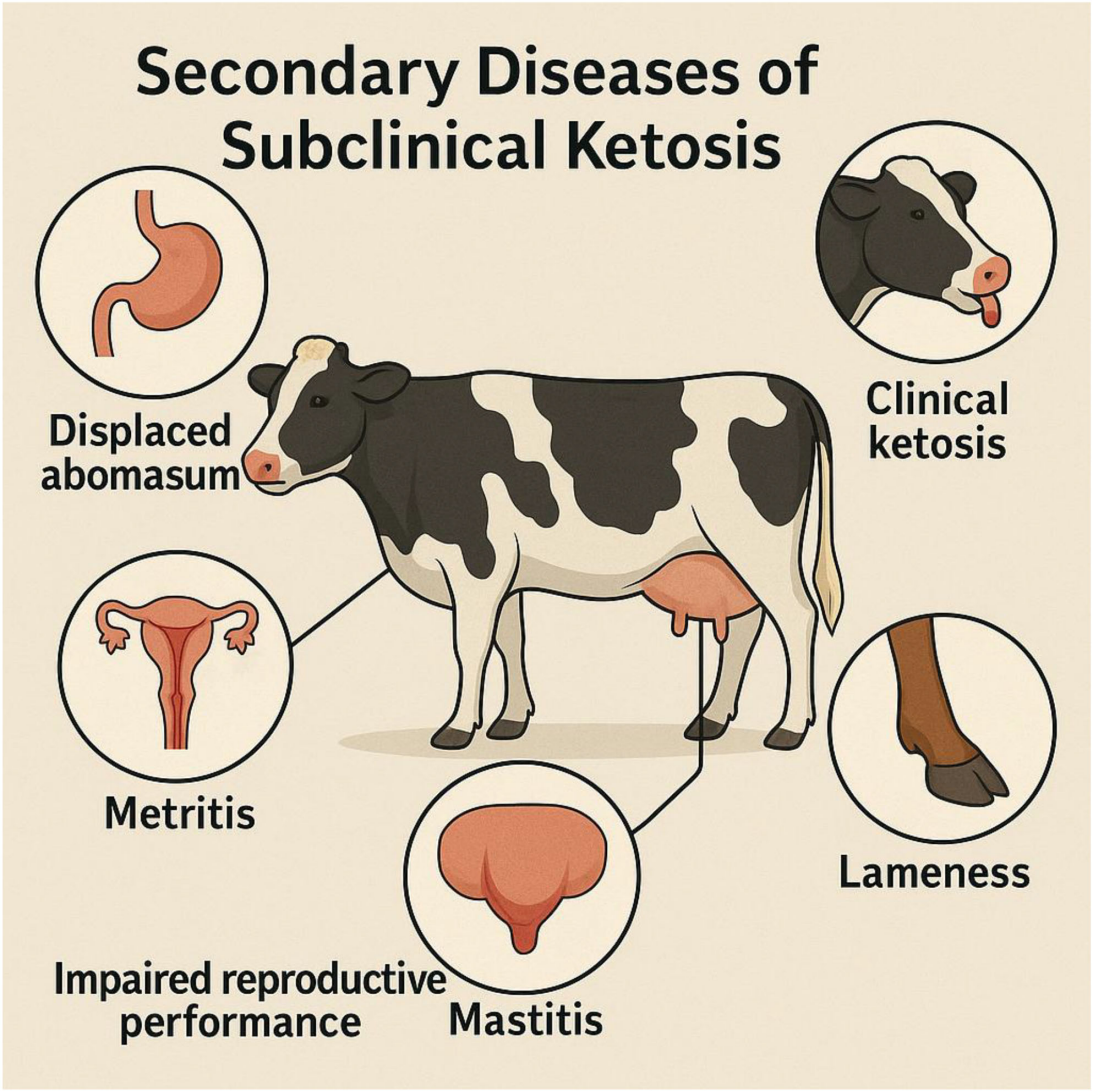
Secondary health and production disorders associated with subclinical ketosis (SCK) in dairy cows. Overview of major postpartum disorders linked to subclinical ketosis during early lactation. Elevated ketone bodies and negative energy balance increase the risk of metabolic, infectious, and reproductive complications, including displaced abomasum, metritis, mastitis, retained placenta, lameness, reduced milk yield, impaired fertility, and increased culling. The figure highlights the important role of SCK as a gateway metabolic disorder influencing overall cow health, productivity, and herd profitability.

Metabolic diseases are common in dairy cows during their transitional period, where they significantly influence the health, productivity, and profitability of a dairy herd. Subclinical ketosis (SCK) is a widespread and economically important metabolic disorder affecting high-producing dairy cows, especially during the early postpartum phase. It leads to decreased performance and is marked by elevated ketone levels without visible clinical signs. As a result, SCK often goes undetected, making it a hidden but costly problem in dairy herds (1–5). Its occurrence ranges from 15 to 60% within the first three weeks of lactation, depending on herd management, nutrition, and monitoring methods (6–8). SCK reduces milk production, with affected cows producing 2–4 kg less milk daily during early lactation (9). It also hampers reproductive performance by causing delayed ovulation, lower conception rates, and longer calving intervals (10). Furthermore, the condition increases the risk of secondary issues such as displaced abomasum, clinical ketosis, metritis, mastitis, and lameness, which leads to higher treatment costs and culling rates (**Figure 1**; 11, 12). The economic loss per case of SCK depends on severity and related complications (13, 14). At the herd level, SCK is an important indicator of how well transition cow management and metabolic health are maintained. Using a blood sample to detect ketone bodies in large dairy herds is not practical due to its intensive procedure and high cost. Therefore, assessment of milk production and urine samples was done for early detection and effective prevention as optimizing productivity, ensuring animal welfare, and supporting the economic sustainability of dairy farms is essential.

Since SCK is a common disease worldwide, reducing the occurrence, severity, and consequences of negative energy balance in the early postpartum period has become a key concern for the dairy industry. The development of quick, on-farm diagnostic tools and improved transition cow management strategies has improved early detection and prevention. However, there is still significant variation in incidence and outcomes across different production systems and geographic regions. Because of the serious implications of SCK for animal health, reproductive performance, and farm profitability, a thorough understanding of its development, diagnosis, risk factors, and control methods is essential. This review aims to gather current knowledge on subclinical ketosis in dairy cows and provide practical insights to improve transition cow health and dairy herd management.

This review synthesizes published literature on subclinical ketosis in dairy cows during the transition period, with a focus on endocrine regulation, hepatic metabolic adaptations, immune–metabolic interactions, and diagnostic approaches. The discussion is primarily centered on dairy production systems and herd-level management contexts. While several metabolic pathways described are conserved across mammalian species, this review does not aim to extrapolate findings beyond dairy cattle, and conclusions should be interpreted within this specific biological and production framework.

Although several reviews have examined metabolic disorders in transition dairy cows, most have focused primarily on nutritional management or epidemiological aspects of ketosis. The present review aims to bridge this gap by integrating endocrine regulation, hepatic metabolic flexibility, immune–metabolic interactions, and emerging diagnostic technologies within a unified conceptual framework. By synthesizing these dimensions, the review provides a mechanistic understanding of subclinical ketosis while also highlighting translational opportunities for early detection and precision herd management.

### 1.1 Literature search strategy

To compile the literature included in this review, a structured search of major scientific databases including Web of Science, Scopus, PubMed, and Google Scholar was conducted. The search covered publications from 2000 to 2025, with emphasis on recent advances in endocrine regulation, metabolic adaptations, and diagnostic approaches related to subclinical ketosis in dairy cows. Keywords used included “subclinical ketosis,” “transition dairy cows,” “β-hydroxybutyrate,” “non-esterified fatty acids,” “endocrine regulation,” “metabolic disorders,” “ketosis diagnostics,” and “precision livestock monitoring.” Both original research articles and relevant review papers published in peer-reviewed journals were considered. Studies focusing specifically on transition dairy cows and metabolic biomarkers associated with ketosis were prioritized.

## 2 Pathophysiology

The transition period, characterized as the time frame from three weeks before calving to three weeks after, is a critical phase for dairy cows, during which they experience considerable metabolic, hormonal, and nutritional adjustments (15–19).

During early lactation, reduced dry matter intake and increased energy demands create a state of negative energy balance (11, 20–23). To compensate, adipose tissue reserves are mobilized, releasing non-esterified fatty acids (NEFAs) into the bloodstream (15, 16, 24, 25). These fatty acids are transported to the liver, where they are oxidized for energy, converted into ketone bodies, or re-esterified into triglycerides. When lipid mobilization exceeds hepatic oxidative capacity, ketone bodies accumulate, predisposing cows to subclinical ketosis (6, 26–28).

**Figure 2:**
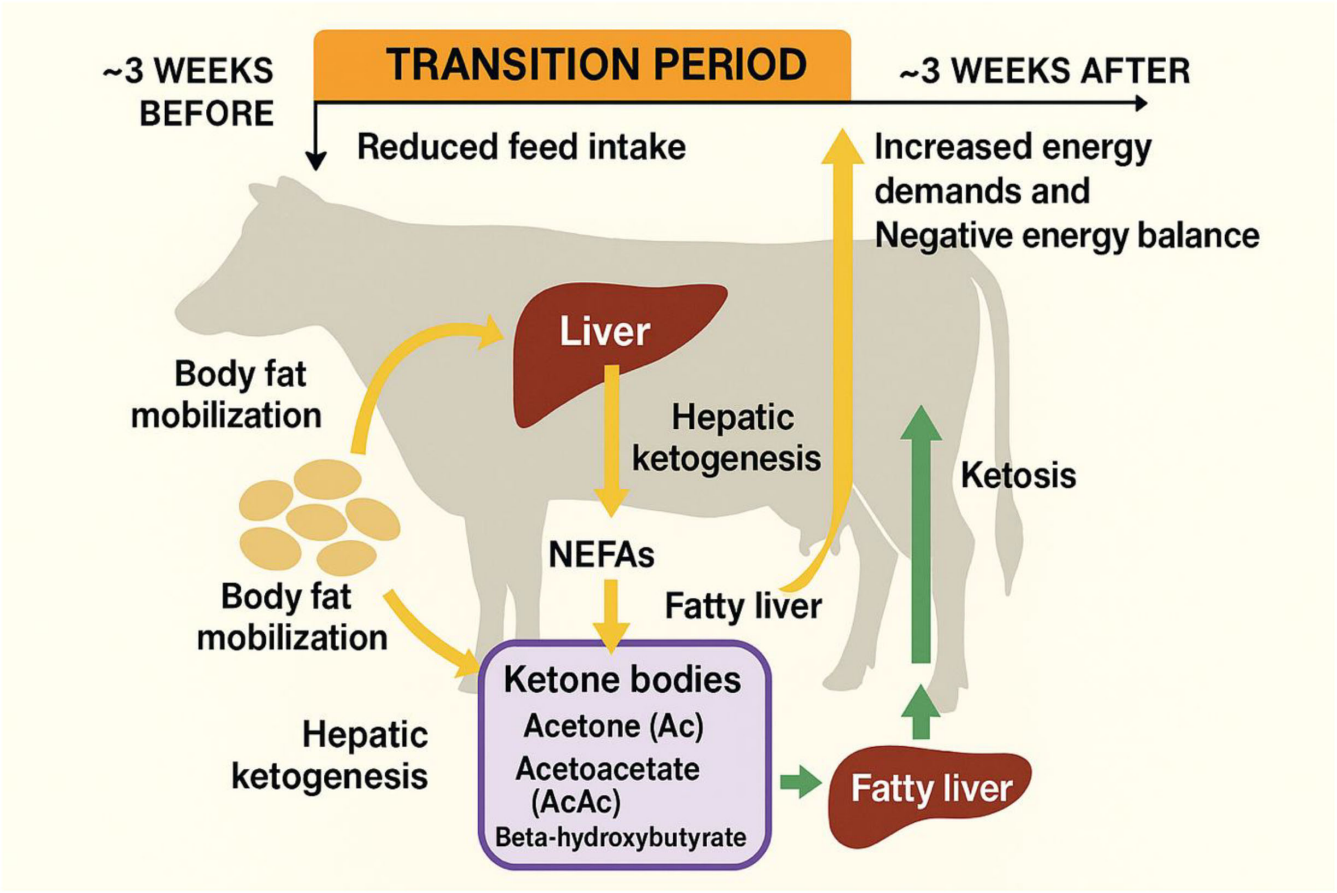
Pathophysiological cascade leading to ketosis in transition dairy cows.

The principal ketone bodies namely acetone (Ac), acetoacetate (AcAc), and β-hydroxybutyrate (BHBA) readily diffuse across cellular membranes. When their production exceeds the cow’s capacity for utilization or excretion, these metabolites accumulate in blood, milk, urine, and other body fluids, leading to ketosis and impaired productivity (29–31). Elevated concentrations of circulating NEFAs and ketone bodies can also impair hepatic metabolic function, reduce feed intake, and compromise immune responses, thereby increasing susceptibility to secondary periparturient diseases (12, 32–35). In severe cases, excessive triglyceride accumulation in hepatocytes leads to hepatic lipidosis (fatty liver), which further disrupts gluconeogenesis and ketone clearance (**Figure 2**).

Schematic illustration of metabolic events during the transition period showing reduced dry matter intake–induced negative energy balance (NEB), enhanced adipose tissue lipolysis, and elevated circulating non-esterified fatty acids (NEFAs). Excess NEFA uptake by the liver overwhelms hepatic oxidative and export capacity, resulting in increased ketogenesis (β-hydroxybutyrate, acetoacetate, acetone) and triglyceride accumulation (fatty liver). The ensuing hyperketonemia and hepatic dysfunction impair gluconeogenesis, suppress feed intake, and compromise immune function, thereby predisposing cows to subclinical and clinical ketosis and associated periparturient disorders.

## 3 Types of ketosis

Ketosis can be categorized into subclinical ketosis (SCK) and clinical ketosis (CK) based on the levels of ketone bodies present in tissues and bodily fluids, along with associated clinical manifestations. Unlike clinical ketosis, which can be recognized through symptoms like decreased feed intake and lethargy, SCK frequently remains unnoticed, despite its significant effects on the health and productivity of cows. Even in the absence of clear clinical signs, cows suffering from SCK often exhibit subtle changes in behavior and physiology, such as reduced rumination, lower milk production, and changes in milk composition—like an increased fat-to-protein ratio—suggesting an imbalance in energy metabolism (36). The early onset of SCK considerably affects productive and reproductive efficiency, as well as overall welfare. Cows that develop SCK in the initial week of lactation tend to produce less milk than those who experience it in the second week (37). Conversely, the financial losses associated with subclinical ketosis can be reduced through the early identification and management of affected cows (38). Additionally, low insulin levels and heightened insulin resistance encourage lipolysis, while increased growth hormone and decreased levels of insulin-like growth factor-1 (IGF-1) drive nutrient distribution towards milk production instead of tissue repair or storage (39). This hormonal environment supports milk production but exacerbates negative energy balance (NEB) and ketones accumulation (40–42).

## 4 Endocrine regulation, hepatic metabolic flexibility, and immuno-metabolic adaptations

While the previous section described the metabolic cascade leading to ketosis, this section focuses on the endocrine mechanisms regulating lipid mobilization and metabolic adaptation during the transition period. The transition period represents a phase of extreme metabolic plasticity during which dairy cows undergo coordinated endocrine and immunometabolic reprogramming to support lactogenesis. Central to this process is a pronounced shift in nutrient partitioning driven by declining dry matter intake and rising glucose demands of the mammary gland, culminating in a sustained negative energy balance (NEB) (15, 16). This metabolic pressure elicits a characteristic endocrine profile characterized by low insulin, elevated growth hormone (GH), suppressed insulin-like growth factor-1 (IGF-1), and increased cortisol concentrations, which collectively promote adipose tissue lipolysis and redirect nutrients toward milk synthesis (26, 39–42).

**Figure 3:**
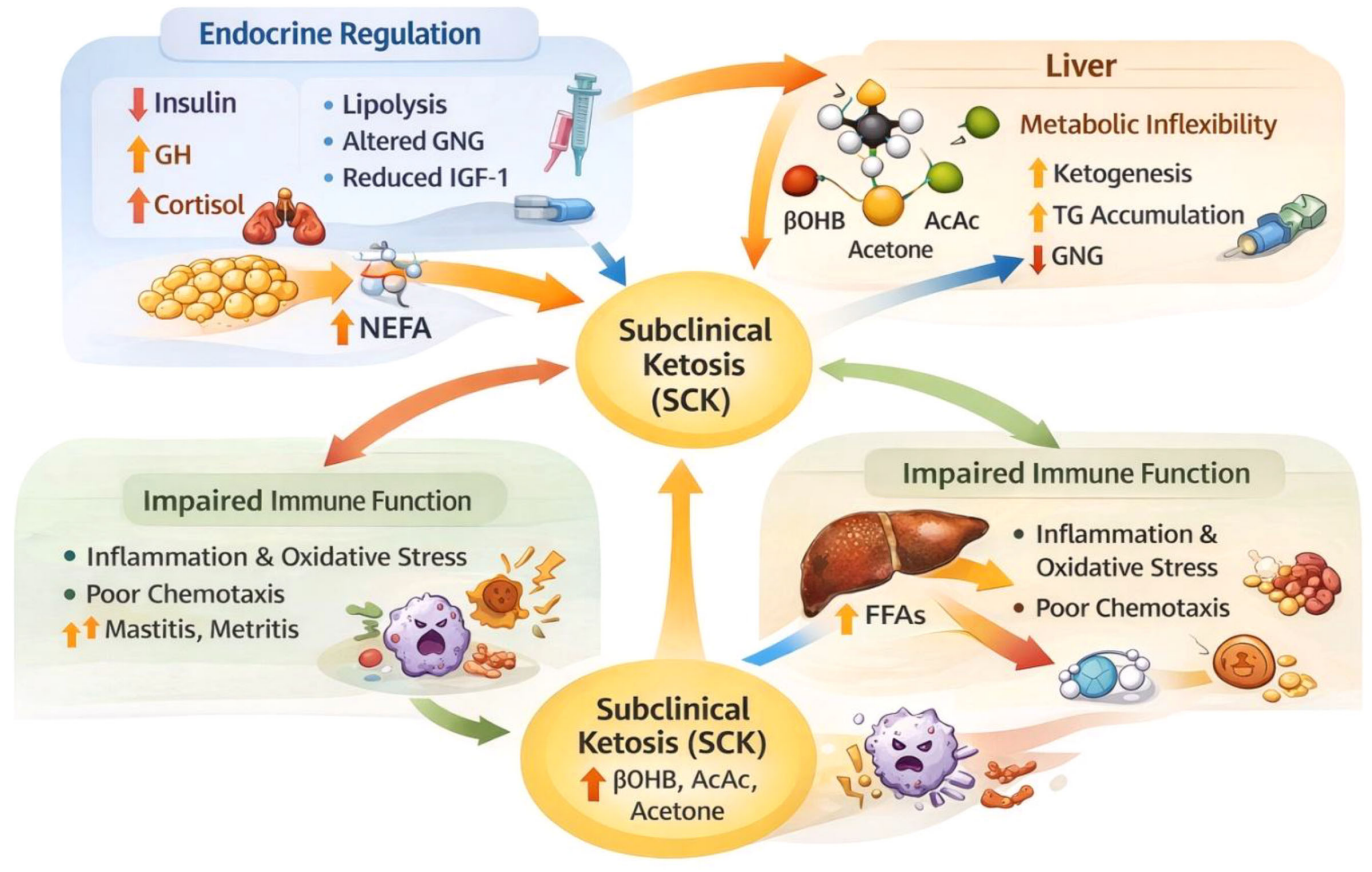
Integrated endocrine–hepatic–immune interactions underlying subclinical ketosis (SCK) in transition dairy cows. Conceptual model illustrating how coordinated endocrine adaptations during early lactation—characterized by decreased insulin and IGF-1 and increased growth hormone and cortisol—promote adipose tissue lipolysis and elevate circulating non-esterified fatty acids (NEFAs). Excessive NEFA influx impairs hepatic metabolic flexibility, leading to increased ketogenesis, triglyceride accumulation, and reduced gluconeogenic capacity. Concurrently, elevated ketone bodies and fatty acids compromise immune cell function and inflammatory regulation, increasing susceptibility to postpartum diseases. Bidirectional feedback among endocrine signaling, hepatic metabolism, and immune responses collectively drives the development and persistence of subclinical ketosis.

Reduced insulin sensitivity during early lactation further enhances the activity of hormone-sensitive lipase in adipose tissue, accelerating lipid mobilization and increasing circulating non-esterified fatty acids (NEFAs). Although this physiological response supports the energetic demands of milk production, excessive lipid mobilization can challenge hepatic metabolic capacity and contribute to metabolic imbalance. Hepatic metabolic flexibility, defined as the liver’s ability to coordinate fatty acid oxidation, gluconeogenesis, triglyceride synthesis, and ketone body production, plays a critical role in maintaining metabolic homeostasis during this period (**Figure 3**; 32). When hepatic metabolic flexibility becomes compromised, triglyceride accumulation and enhanced ketogenesis can occur, increasing the risk of metabolic disorders such as subclinical ketosis.

At the molecular level, insulin resistance during early lactation involves alterations in intracellular signaling pathways associated with the insulin receptor. Reduced phosphorylation of insulin receptor substrate-1 (IRS-1) and downstream modulation of phosphatidylinositol-3-kinase (PI3K) and AKT signaling pathways decrease insulin-mediated glucose uptake in peripheral tissues while promoting adipose tissue lipolysis (43, 44). These cellular adaptations ensure that glucose is preferentially directed toward the mammary gland for lactose synthesis, but they also contribute to enhanced lipid mobilization during negative energy balance in transition dairy cows (45, 46).

Adipose tissue also functions as an endocrine organ through the secretion of adipokines that influence metabolic regulation. For example, adiponectin plays an important role in regulating hepatic lipid metabolism and insulin sensitivity, whereas leptin is involved in the control of feed intake and overall energy balance (47, 48). In dairy cows, alterations in adipokine signaling and adipose tissue metabolic activity during the transition period have been associated with changes in insulin responsiveness and lipid mobilization (49, 50). Proteomic studies of bovine adipose tissue have further demonstrated differential expression of proteins involved in lipid metabolism, energy utilization, and metabolic efficiency, highlighting the role of adipose tissue as an active metabolic regulator during lactation (51). Consequently, dysregulation of adipokine signaling may therefore influence the magnitude of lipid mobilization and metabolic stress during the transition period.

Beyond endocrine regulation, metabolic stress during early lactation is closely linked to immune function. Elevated concentrations of circulating fatty acids and ketone bodies can impair leukocyte oxidative responses, reduce neutrophil chemotaxis, and alter cytokine signaling pathways, reflecting the metabolic constraints placed on immune function during early lactation (33). Pro-inflammatory cytokines such as tumor necrosis factor-α (TNF-α) and interleukin-6 (IL-6) interact with metabolic signaling pathways and may further exacerbate insulin resistance and hepatic metabolic stress. This interaction between metabolic and immune pathways, often described as immunometabolic regulation, contributes to increased susceptibility to postpartum diseases including metritis, mastitis, displaced abomasum, and retained placenta, which are closely associated with poorer reproductive outcomes and increased culling risk (2, 12).

Because these endocrine, metabolic, and immune pathways are tightly interconnected, disruption of this coordinated regulatory network can predispose transition cows to metabolic disorders such as subclinical ketosis. Understanding these integrated mechanisms and their physiological consequences is therefore essential for developing improved diagnostic strategies and management interventions aimed at maintaining metabolic health in modern dairy production systems.

Together, these findings highlight that transition cow metabolism operates as an integrated endocrine–hepatic–immune regulatory network that determines metabolic resilience during early lactation. Furthermore, current evidence demonstrates that SCK arises from a complex interplay of endocrine-driven lipolysis, constrained hepatic metabolic flexibility, and immune suppression.

## 5 Epidemiology

In recent years, there has been a shift in the occurrence of SCK, where greater frequency was observed between 3- and 5-days post-partum compared to 14- and 21-days post-partum previously, which may be attributed to the intense genetic selection pressure for milk production (52). The occurrence of SCK is notably greater than that of CK (11). This condition usually becomes apparent during the initial 2 to 3 weeks after giving birth (6, 15). Although the disorder does not present with observable clinical symptoms, its high incidence and broad distribution render it a major issue in both intensive and extensive dairy farming systems globally. In a herd, the identification of SCK in 10% of cows should be considered an alarming level (53).

The prevalence of Subclinical Ketosis (SCK) varies based on the population studied, the diagnostic criteria applied, and the detection method employed. A global prevalence rate of 22.7% has been recorded, while in India, the rate is slightly higher at 23.50% (8, 54). Brunner et al. (55) reported a global prevalence of 24.1% ranging from 8.3% to 40.1%. In herds where metabolic profiling is regularly performed, the prevalence usually falls between 15% and 60%, with some regions seeing herd-level rates exceeding 80% (6, 12). A significant study involving over 8,900 cows from 100 commercial dairy herds in Canada indicated that the SCK prevalence was 21.8% during the first 35 days post-calving (1). Comparable research in the United States and Europe has shown prevalence rates between 19 and 45%, with variations attributable to factors like parity, season, nutrition, and housing conditions (2, 12, 36). Multiparous cows face a higher risk of SCK due to their increased milk output and heightened body fat mobilization (10). Seasonal differences also affect SCK prevalence, with elevated rates typically occurring in winter and early spring, likely linked to variations in diet, feed intake, and environmental stressors (20). Furthermore, cows with a body condition score (BCS) of 3.5 or higher at calving are at an increased risk of developing SCK due to significant lipid mobilization after giving birth (56).

Geographically, this condition is prevalent, with instances of subclinical ketosis (SCK) reported from dairy farming areas throughout North America, Europe, South America, and Asia. However, differences in diagnostic methods, sampling techniques, and herd management practices hinder straightforward comparisons between research findings. The epidemiology of subclinical ketosis indicates that it is a significant and widely occurring issue in contemporary dairy herds worldwide. Its prevalence is affected by factors such as parity, body condition, nutritional status, season, and management system, highlighting the necessity for specific prevention and monitoring strategies tailored to the unique conditions of each herd.

## 6 Diagnoses

Early detection of metabolic disorders is essential for effective herd management and for preventing the progression to clinical disease. Subclinical ketosis (SCK) is primarily diagnosed by detecting elevated concentrations of ketone bodies in biological fluids, including blood, milk, and urine. Laboratory-based biochemical analyses are traditionally used for this purpose; however, these methods are labor-intensive, time-consuming, and require specialized equipment and trained personnel. To facilitate rapid detection under field conditions, several commercial diagnostic kits have been developed that allow simple, rapid, and reliable identification of ketosis in dairy cows (57).

For herd level monitoring, screening a representative subset of cows between 5 and 15 days postpartum is commonly recommended. If more than 15–20% of cows test positive, the herd is considered to have a subclinical ketosis problem requiring management intervention (6). Diagnosis of SCK mainly relies on quantifying ketone bodies, particularly β-hydroxybutyrate (BHBA) in blood, milk, or urine (1, 2). A strong correlation between blood and milk ketone body concentrations has been reported, supporting the use of milk-based tests for herd screening (58). Diagnostic efforts are typically focused on cows during early lactation, particularly between 3 and 21 days postpartum, when the risk of metabolic disorders is the highest.

Robust identification of SCK requires metabolic biomarkers that reliably reflect the underlying endocrine–metabolic imbalance associated with negative energy balance. Non-esterified fatty acids (NEFAs) serve as an early indicator of excessive lipid mobilization, and prepartum concentrations exceeding 0.26 mmol/L have been associated with an increased risk of postpartum metabolic diseases (59). However, the most widely accepted biomarker for diagnosing SCK is blood β-hydroxybutyrate (BHBA), which is considered the diagnostic gold standard. Diagnostic thresholds for blood BHBA generally range between 1.2 and 1.4 mmol/L, with reported sensitivities and specificities exceeding 80% in many field studies (2, 53). Nevertheless, the predictive value of BHBA measurements may vary depending on sampling time relative to calving and herd management conditions. Consequently, increasing attention has been directed toward predictive biomarkers capable of identifying cows at risk before the development of hyperketonemia. Elevated prepartum NEFA concentrations, changes in milk fatty acid profiles, and multi-marker metabolic panels have shown promise as early indicators of metabolic imbalance during the transition period.

Several cow-side diagnostic tools have been developed to enable rapid detection of ketone bodies under field conditions. Portable handheld meters such as Precision Xtra™ and Nova Vet™ allow direct measurement of blood BHBA concentrations with high accuracy and convenience (60). Studies have demonstrated that handheld ketometers can provide reliable results comparable to laboratory-based BHBA analyses (61). Although blood-based measurements provide high diagnostic accuracy, they remain invasive and labor-intensive when applied across large herds.

Milk-based tests represent a practical and non-invasive alternative for herd-level screening. Milk ketone testing, particularly for BHBA or acetone concentrations, can be incorporated into routine milk recording programs. Mid-infrared (MIR) spectroscopy is widely used in dairy herd improvement programs to estimate milk BHBA concentrations (36). A milk BHBA concentration exceeding 0.10–0.15 mmol/L is commonly used as an indicator of SCK (11). Although milk-based tests may exhibit slightly lower sensitivity and specificity than blood BHBA measurements, improvements in analytical techniques such as Fourier-transform infrared (FTIR) spectroscopy have enhanced the accuracy of early SCK risk estimation (62).

Urine-based detection methods are also used as a simple and inexpensive screening approach. Ketone test strips (e.g., Ketostix™) detect acetoacetate in urine samples and provide rapid results under field conditions. However, urine tests generally exhibit lower sensitivity and may fail to detect early cases of SCK because ketone excretion in urine varies depending on hydration status and the timing of sample collection (38).

These diagnostic approaches collectively illustrate the need for integrated, multi-biomarker monitoring strategies. Because repeated monitoring with minimal animal stress is desirable in commercial dairy systems, non-invasive diagnostic approaches are increasingly preferred. Milk MIR spectroscopy enables population-level screening of ketone signatures and is now integrated into routine herd testing programs, although its diagnostic accuracy remains slightly lower than that of blood-based biomarkers (36, 63). In addition, emerging biosensor technologies including breath acetone sensors, saliva-based BHBA assays, quantum-dot microfluidics devices, and wearable metabolic monitoring systems offer new opportunities for continuous, real-time metabolic monitoring (64, 65). These innovations align with the rapid development of precision livestock farming, enabling earlier detection, improved treatment timing, and more effective herd-level management of metabolic disorders.

The choice of threshold depends on the biomarker and sample type. The commonly used thresholds are elaborated in **Table 1**.

**Table 1:** Commonly used thresholds of various ketone bodies in different body fluids for categorization of subclinical ketosis (SCK) status of the dairy animals.

Sample type	Biomarker	Threshold indicating SCK	References
Blood	BHBA	≥ 1.20-1.40 mmol/L	2, 6, 11, 53, 60, 66, 67
Milk		≥ 0.10-0.15 mmol/L	68, 69; 70
Urine		≥ 40–80 mg/dL	6, 71
Blood	Acetoacetate	≥30-50 µmol/L	72, 73
Milk		≥100-190 µmol/L	38
Urine		≥ 80–120 mg/dL	71, 73
Blood	Acetone	≥ 70-100 µmol/L	72, 74–76
Milk		≥120-200 µmol/L	58, 72, 75, 77–80

During subclinical ketosis (SCK), there is an increase in the percentage of milk fat and a decrease in the percentage of protein. Therefore, alterations in milk composition, such as a rise in the milk fat-to-protein ratio greater than 1.4, may indicate SCK (81), which exhibits a sensitivity of approximately 58% and specificity of 69%, even though the correlation between blood BHBA and the fat-to-protein ratio is low (r = 0.17; 82). Caldeira et al. (63) observed that before the diagnosis of ketosis, there is often a more significant increase in milk acetone than in milk BHBA. So, this fat-to-protein ratio provides lower accuracy than the inclusion of Ac and BHBA in milk for the detection of SCK (83). Furthermore, a body condition score (BCS) of 3.5 or higher at calving, along with significant weight loss postpartum, can corroborate a presumptive diagnosis (56). A non-esterified fatty acid in blood serum was also used for the detection of SCK with a threshold of >0.26 mmol/L (59). For an accurate diagnosis of subclinical ketosis, a combination of biochemical analyses, timely sample collection, and assessment of risk factors is crucial. Blood BHBA testing is still considered the gold standard, while milk and urine tests can assist in the screening of larger herds. Early identification allows for precise interventions and can help minimize potential health issues and production declines.

Despite considerable progress in biomarker-based detection, several challenges remain in the accurate diagnosis of subclinical ketosis. Reported threshold values for BHBA and NEFA vary among studies due to differences in sampling time, analytical methods, and herd management conditions. In addition, while blood BHBA remains the diagnostic gold standard, large-scale herd monitoring using blood samples to detect ketone bodies in large dairy herds is often impractical due to labor and cost constraints. Non-invasive approaches such as milk MIR spectroscopy or wearable sensor technologies show promise but currently exhibit variable diagnostic accuracy. Therefore, integrating multiple biomarkers with precision-livestock monitoring systems may provide more reliable strategies for early detection and management of subclinical ketosis in dairy production systems, highlighting the need for integrated, multi-parameter diagnostic frameworks.

### 6.1 Diagnostic methods for subclinical ketosis

**Figure 4:**
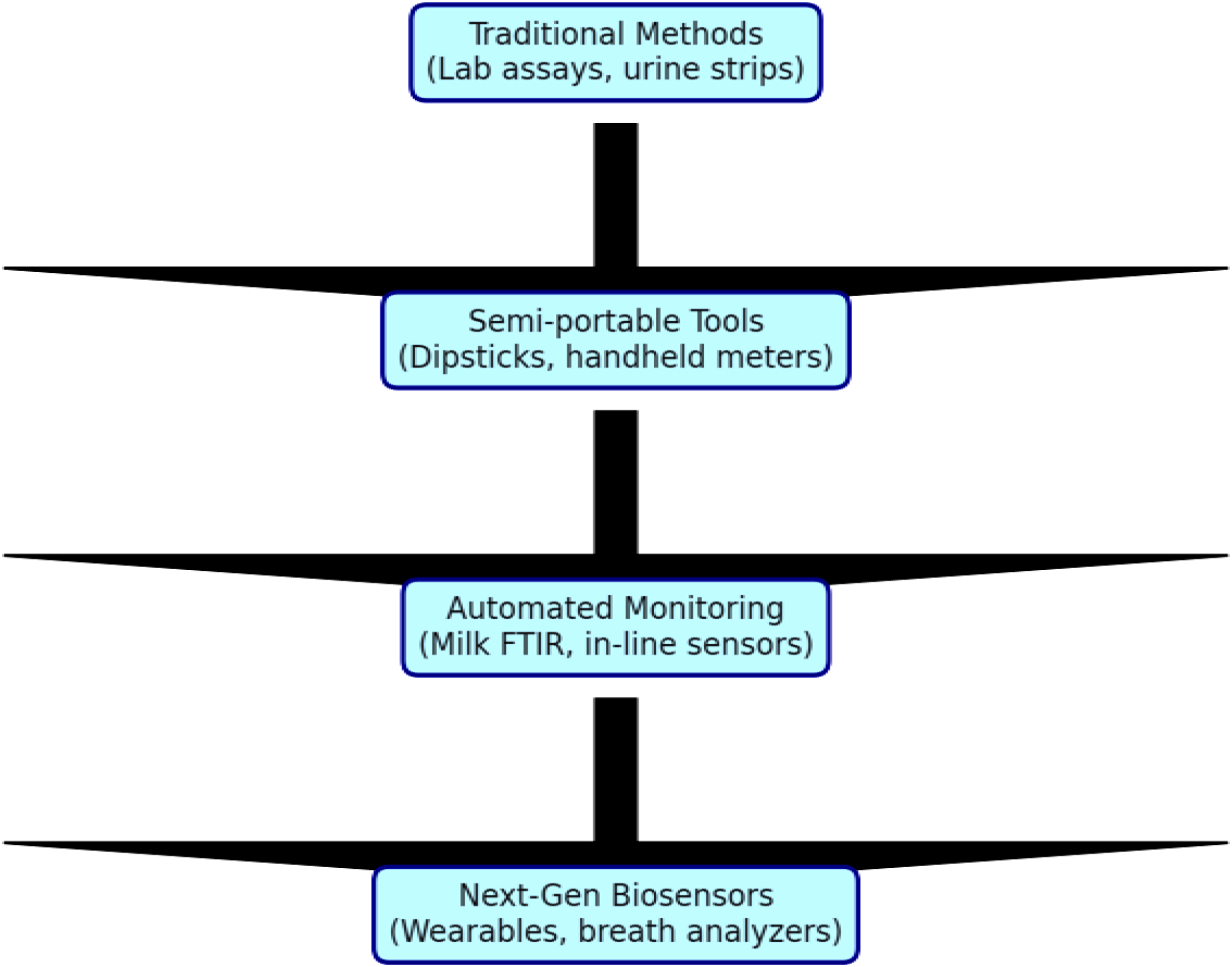
Graphical flowchart to illustrate the progression from traditional methods to modern biosensor-based approaches to diagnosis of subclinical ketosis. The flowchart depicts the progression from conventional laboratory-based diagnostic methods to modern cow-side and biosensor-driven technologies for the detection of subclinical ketosis. Traditional blood, milk, and urine ketone measurements are contrasted with advanced approaches such as handheld β-hydroxybutyrate meters, milk mid-infrared (MIR/FTIR) spectroscopy, breath acetone analysis, and emerging wearable or microfluidic biosensors. The figure emphasizes improvements in rapidity, non-invasiveness, sensitivity, and herd-level applicability, supporting earlier detection and precision management of SCK.

**Figure 5:**
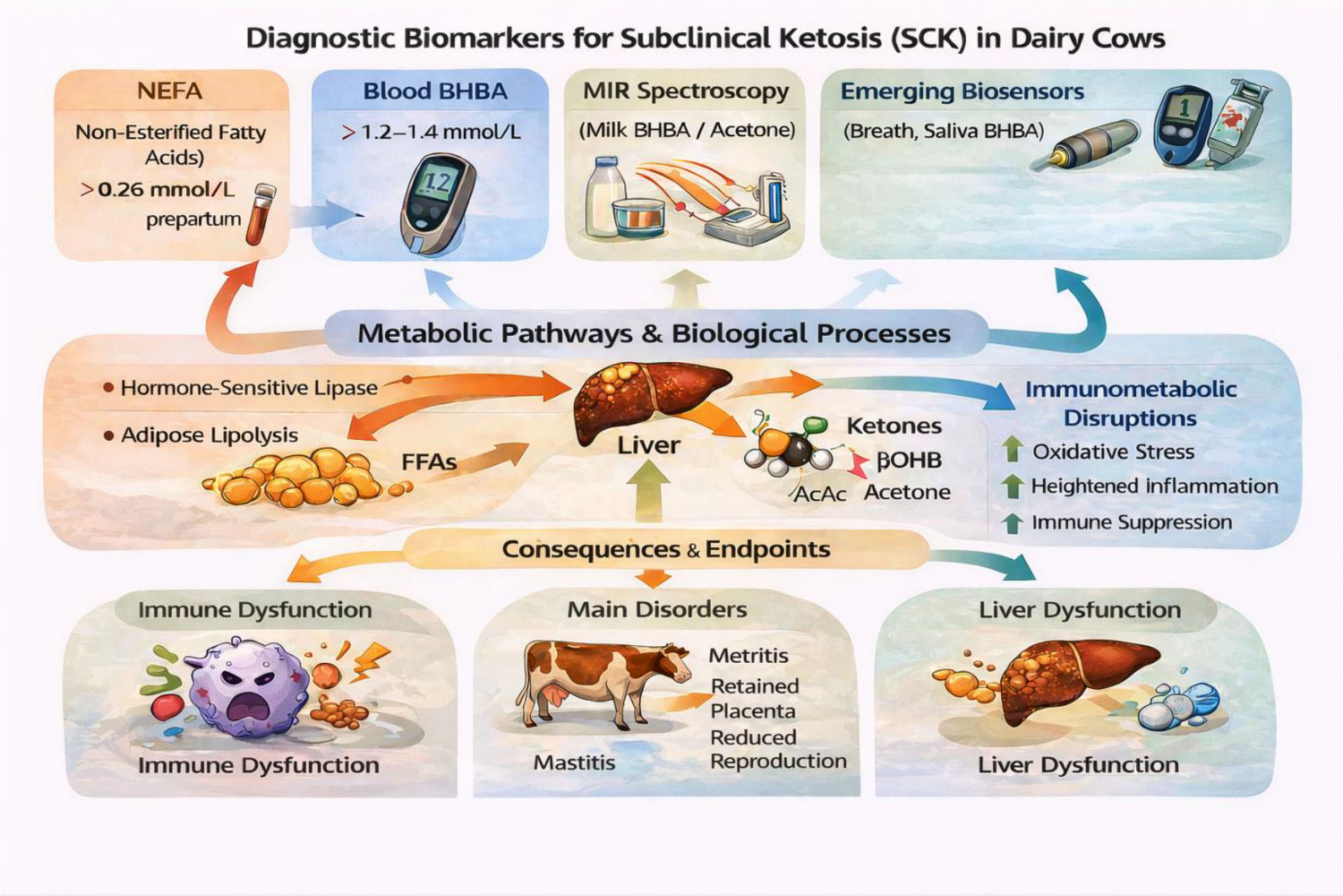
Diagnostic biomarkers and metabolic pathway interactions associated with subclinical ketosis (SCK) in dairy cows. Schematic representation of key metabolic pathways and diagnostic biomarkers involved in the development of subclinical ketosis during the transition period. Enhanced adipose tissue lipolysis elevates circulating non-esterified fatty acids (NEFAs), which are taken up by the liver and directed toward β-oxidation, ketogenesis, or triglyceride storage. Accumulation of ketone bodies, particularly β-hydroxybutyrate (BHBA), in blood, milk, urine, and breath serves as the primary diagnostic indicator of SCK. The figure highlights the interrelationships between endocrine regulation, hepatic metabolic flexibility, and measurable biomarkers used for early detection and monitoring of SCK at the cow and herd levels.

This document presents a comparative overview of diagnostic methods for Subclinical Ketosis (SCK) in dairy cows, highlighting their principles, advantages, limitations, and evolution toward point-of-care systems (**Table 2**). It also includes a graphical flowchart to illustrate the progression from traditional methods to modern biosensor-based approaches (**Figures 4**, **5**).

**Table 2:** A comparative overview of diagnostic methods for Subclinical Ketosis (SCK) in dairy cows.

Method	Principle	Advantages	Limitations	Point-of-care evolution	References
Blood BHBA Testing	Direct measurement of β-hydroxybutyrate in blood	Gold standard; high sensitivity & specificity; rapid with handheld meters	Invasive (venipuncture); costlier; labor-intensive for herd	Handheld BHBA meters (cow-side, immediate results)	84, 85
Milk Ketone Testing	Detection of ketones (acetone, BHBA) in milk via strips, dipsticks, or spectroscopy	Non-invasive; easy sampling; adaptable to milking routines	Less sensitive than blood; lag in detection; possible false negatives	Dipstick/strip kits; in-line milk sensors in robotic milking	36, 61, 74, 86
Urine Ketone Testing	Reagent strips detect acetoacetate/acetone in urine	Cheap; simple; quick screening	Requires urine collection; stress to animal; low sensitivity; variable results	Limited—mostly dipstick-based; less practical for herd monitoring	87, 88
Milk FTIR Spectroscopy	Indirect estimation of ketone bodies from routine milk samples	Automated; uses existing milk samples; scalable for herd monitoring	Indirect measurement; lower accuracy; often off-farm lab analysis	Being integrated into on-farm milk analyzers	63, 89
Other Biomarkers (NEFA, Breath Ketones, etc.)	Measurement of predictive markers (NEFA in blood, ketones in breath/saliva)	May allow earlier risk prediction; some non-invasive options	Require specialized equipment; not yet widely available	Emerging biosensors (breath analyzers, wearables, saliva/blood sensors)	64, 65, 90–93

## 7 Economics and productivity impact

Metabolic diseases are among the most important diseases from an economic point of view in the dairy cattle industry. Detection of the disease is needed as there is an increase in the demand for a healthy product from consumers and also for early treatment to prevent further loss. Subclinical ketosis (SCK) has significant economic and productivity consequences for dairy operations, despite its lack of clinical signs. The metabolic disruption associated with SCK leads to decreased milk yield, reproductive inefficiencies, and increased incidence of secondary diseases, all of which contribute to substantial financial losses at both the cow and herd levels (2, 14).

Cows diagnosed with SCK in early lactation have been shown to produce 1.5 to 3.5 kg less milk per day compared to healthy cows (1, 6). This reduction may persist for several weeks, resulting in cumulative losses of up to 80 to 150 kg of milk per cow per lactation. In addition to quantity, SCK can negatively affect milk composition, particularly by increasing the fat-to-protein ratio, which may result in penalties under milk pricing schemes (81). SCK is associated with delayed ovarian cyclicity, lower conception rates, and longer days open, all of which reduce reproductive efficiency (10). Cows with SCK are approximately 1.5 times more likely to be culled for reproductive failure (9). Prolonged calving intervals and reduced pregnancy rates translate into increased insemination costs and lost calving opportunities. Cows with SCK have significantly increased odds of developing displaced abomasum, retained placenta, metritis, mastitis, and lameness (11, 12). The risk of displaced abomasum is up to five times higher in SCK-affected cows (6). These diseases incur both direct veterinary expenses and indirect losses due to reduced performance and premature culling. SCK increases the likelihood of early culling due to poor health and reproductive failure. Cows with persistent or repeated SCK episodes are also at higher risk of mortality, especially if concurrent diseases occur (2). The economic loss per case of SCK varies depending on herd management, milk price, and regional disease treatment costs. Estimates range from $78 to $289 USD per affected cow, considering lost milk production, delayed reproduction, increased veterinary costs, and elevated culling rates (13, 14). At the herd level, these costs can amount to thousands of dollars annually, especially in high-prevalence situations. Implementing effective monitoring and prevention strategies can reduce the financial burden and improve overall herd performance.

## 8 Prevention and management

Prevention and management of subclinical ketosis (SCK) is essential to maintaining dairy herd health, productivity, and economic viability. As SCK typically develops during the transition period—particularly within the first 2 to 3 weeks postpartum—strategies must focus on improving energy balance, reducing excessive lipid mobilization, and enhancing liver function (15, 16). Preventive measures involve a multifaceted approach combining nutritional management, monitoring, and, where necessary, therapeutic interventions.

Providing well-formulated prepartum and postpartum diets is central to SCK prevention. Prepartum diets should aim to maintain a moderate body condition score (BCS 3.0–3.5) and encourage adequate dry matter intake (DMI) (56). Postpartum diets must be energy-dense, highly palatable, and balanced for fiber, protein, and micronutrient to support the cow’s increasing energy demands. Incorporation of glucogenic precursors (e.g., propylene glycol) can improve energy status and reduce BHBA levels (67). Adequate rumen-degradable starch supports propionate production, a key precursor for hepatic gluconeogenesis (94). Supplementing with niacin has been shown to reduce lipolysis and ketogenesis, though results are variable (95). Monensin, an ionophore, improves energy metabolism by enhancing ruminal propionate production and has been shown to reduce the incidence of SCK (96, 97). Routine metabolic profiling during the transition period enables early detection and targeted intervention. Testing blood BHBA concentrations between 5 and 15 days postpartum can help identify at-risk animals before clinical signs or secondary diseases appear (6). Overconditioned cows (BCS ≥3.75 at calving) are at greater risk of SCK due to excessive fat mobilization. Maintaining optimal BCS (3.0–3.5) and minimizing body fat accumulation during the dry period is critical (56). Providing comfortable housing, minimizing stress, ensuring adequate bunk space (≥60 cm/cow), and maintaining cow hierarchy during the periparturient period also help to sustain DMI and reduce risk (98). In cows diagnosed with SCK, propylene glycol is the treatment of choice. Administered orally at 300–500 mL/day for 3–5 days, it serves as a direct gluconeogenic substrate and helps reduce BHBA levels (2). Cascone et al. (7) reported that the treatment with propylene glycol was most effective within the first 7 days of lactation (76.5%) and had an increase in milk yield and milk quality. In severe cases, intravenous glucose or corticosteroids (e.g., dexamethasone) may be used under veterinary supervision, although this is more common in clinical ketosis. Preventing SCK at the herd level requires coordinated management of nutrition, reproduction, health monitoring, and cow flow. Effective prevention and management of subclinical ketosis require a proactive, integrated approach centered on transition cow nutrition, early detection, and cow comfort. By reducing the incidence of SCK, dairy producers can improve milk yield, reproductive performance, and overall herd profitability.

## 9 Recent advances and future directions

Subclinical ketosis (SCK) remains an important area of research because of its substantial effects on the health, productivity, and economic performance of dairy cows. Routine testing of all cows in a herd using standard blood examination methods is often impractical due to financial and logistical constraints. Recent advances have improved our understanding of the molecular mechanisms, diagnostic approaches, and precision management strategies associated with SCK. Future research increasingly focuses on integrating omics technologies, machine learning approaches, and advanced nutritional strategies to improve prevention, early detection, and management of metabolic disorders in dairy cattle.

Recent studies have expanded insights into the pathophysiology of SCK at cellular and molecular levels. Transcriptomic and metabolomic analyses have identified key genes and metabolic pathways involved in lipid mobilization, hepatic oxidative stress, and inflammatory responses during ketosis (99, 100). These findings may facilitate the development of biomarkers for earlier and more precise detection of metabolic imbalance. The development of biosensors and cow-side digital meters for ketone bodies enables faster, more frequent, and less invasive monitoring of metabolic status. Emerging technologies include wearable sensors that continuously monitor physiological indicators such as rumination activity, locomotor behavior, and body temperature, which can be integrated with blood or milk ketone data to predict ketosis risk before clinical signs appear (101, 102).

Precision feeding systems also allow real-time adjustment of nutrient supply according to individual cow requirements, helping to maintain energy balance and reduce ketosis incidence (103). Nutritional strategies involving rumen-protected amino acids, antioxidants, and targeted micronutrients are being optimized using individualized feeding algorithms (104). In addition, genomic selection tools are increasingly being explored to breed cows with improved metabolic resilience. Identifying genetic markers associated with reduced susceptibility to SCK may enhance herd health and productivity across generations (105). Integration of metabolic traits into breeding programs therefore represents a promising long-term strategy for improving metabolic robustness in dairy cattle.

Machine learning approaches are increasingly being applied to large datasets derived from on-farm sensors, metabolic profiles, and herd management records to develop predictive algorithms for assessing SCK risk and guiding management decisions (106). These approaches enable predictive modeling without requiring predefined relationships between variables (107). Logistic regression and other predictive models incorporating variables such as milk fat-to-protein ratio, milk acetone and BHBA concentrations, lactose percentage, lactation number, and days in milk have shown promise for identifying cows at risk of developing SCK. In addition, microbiome research is exploring the role of ruminal and gut microbial communities in energy metabolism and ketosis development, with the aim of developing microbiome-based interventions or probiotic strategies (108).

Future research should prioritize early identification of cows at risk of metabolic imbalance during the prepartum period. Advances in genomic selection and genetic marker identification may help identify animals with improved metabolic resilience. Integrative systems biology approaches combining metabolomics, transcriptomics, and microbiome profiling are also expected to improve understanding of complex endocrine–metabolic interactions during the transition period. In addition, multi-marker diagnostic panels incorporating NEFA, BHBA (109, 110), inflammatory mediators, and behavioral indicators may offer improved predictive performance compared with single-biomarker approaches.

The rapid progress in molecular tools, sensor technologies, and data analytics provides significant opportunities for improving the detection and management of SCK. However, several knowledge gaps remain in understanding and managing subclinical ketosis in modern dairy production systems. Future research should focus on identifying early predictive biomarkers capable of detecting metabolic imbalance before the onset of hyperketonemia. Integration of multi-omics approaches, including metabolomics, transcriptomics, and microbiome profiling, may provide deeper insights into the regulatory mechanisms underlying metabolic adaptation during the transition period. Furthermore, large-scale validation of wearable biosensors and real-time monitoring technologies under commercial farm conditions is required. Development of robust machine-learning models integrating physiological, metabolic, and behavioral data also represents an important frontier for precision dairy management. Future strategies will likely emphasize early, individualized detection and intervention by integrating genetics, nutrition, and precision monitoring technologies to improve the metabolic resilience and productivity of dairy cows in increasingly complex production systems.

## 10 Conclusions

Conceptually, transition cow metabolism can be viewed as an adaptive endocrine network in which reduced insulin signaling, elevated growth hormone activity, and increased lipid mobilization interact with hepatic metabolic capacity and immune function. When this adaptive system becomes dysregulated due to excessive lipid mobilization or inadequate nutritional support, metabolic imbalance develops and predisposes cows to subclinical ketosis. Understanding this integrated endocrine–metabolic framework is essential for developing improved prevention strategies and precision monitoring tools for dairy herd management.

Subclinical ketosis (SCK) therefore emerges from disruptions in this integrated endocrine–hepatic–immune regulatory network during the transition period of dairy cows. Alterations in insulin signaling, increased growth hormone activity, and enhanced lipid mobilization create metabolic conditions that predispose cows to excessive ketone body production. When hepatic metabolic flexibility becomes constrained, accumulation of ketone bodies and triglycerides can disrupt metabolic homeostasis and increase susceptibility to postpartum diseases. These findings emphasize that SCK should be viewed as an integrated endocrine–hepatic–immune metabolic disorder rather than a single metabolic imbalance. Understanding these interconnected endocrine–metabolic processes is therefore essential for improving metabolic health during early lactation.

Furthermore, advances in biomarker discovery, precision monitoring technologies, and systems-level approaches are improving the early detection and management of SCK in dairy production systems. Integration of metabolic biomarkers, wearable sensor technologies, and data-driven predictive models offers new opportunities for proactive herd management. Future research integrating multi-omics approaches, machine learning, and precision nutrition strategies will be critical for developing more effective prevention and intervention programs. Ultimately, improving our understanding of endocrine–metabolic adaptation during the transition period will enhance dairy cow health, productivity, and sustainability of modern dairy systems. Together, these insights provide a framework for predictive metabolic monitoring in transition cows.
